# Prediction of prevalence of type 2 diabetes in Rwanda using the metropolis-hasting sampling

**DOI:** 10.4314/ahs.v21i2.28

**Published:** 2021-06

**Authors:** Angelique Dukunde, Jean Marie Ntaganda, Juma Kasozi, Joseph Nzabanita

**Affiliations:** 1 University of Rwanda, College of Business and Economics, African Center of Excellence in Data Science (ACE-DS); 2 University of Rwanda, College of Science and Technology, School of Science, Department of Mathematics; 3 Makerere University, College of Natural Sciences, Department of Mathematics

**Keywords:** Non communicable diseases, type 2 diabetes, Markov Chain Monte Carlo method, Metropolis-Hasting method, Transition probabilities

## Abstract

In this work, we predict the prevalence of type 2 diabetes among adult Rwandan people. We used the Metropolis-Hasting method that involved calculating the metropolis ratio. The data are those reported by World Health Organiation in 2015. Considering Suffering from diabetes, Overweight, Obesity, Dead and other subject as states of mathematical model, the transition matrix whose elements are probabilities is generated using Metropolis-Hasting sampling. The numerical results show that the prevalence of type 2 diabetes increases from 2.8% in 2015 to reach 12.65% in 2020 and to 22.59% in 2025. Therefore, this indicates the urgent need of prevention by Rwandan health decision makers who have to play their crucial role in encouraging for example physical activity, regular checkups and sensitization of the masses.

## Introduction

Diabetes mellitus commonly refers to as diabetes, is a group of diseases that affect how the body uses blood sugar known as glucose. It is a non communicable disease (NCD) and chronic disease caused by inherited and/or acquired deficiency in production of insulin by the pancreas, or by the ineffectiveness of the insulin produced. This deficiency damages many of the body systems, in particular the blood vessels and nerves. Some of its symptoms include frequent urination and excessive drinking. Others are increased hunger, unexplained weight loss, fatigue, irritability, blurred vision, slow-healing sores, frequent infections, such as gums or skin infections and vaginal infections. In 1999; the World Health Organization (WHO) subdivided diabetes mellitus into four main groups1:

- Type 1 which affects primarily young children. It occurs when the pancreas' failure to produce sufficient insulin;
- Type 2 diabetes (T2D) result when cells body fail to respond to insulin properly and the primary causes are excessive body weight and not enough exercise;
- Gestational diabetes Mellitus (GDM) that occurs when pregnant women who does not have any previous history of diabetes develop high blood sugar levels. It is similar to type 2 diabetes, but usually after the woman give birth it can be resolved. A women with gestational diabetes are at high risk for developing type 2 diabetes later in their life;
- Specific types (heterogenic group), it caused by genetic defects in beta-cell function, in insulin action, genetic syndromes associated with diabetes and diabetes secondary to other conditions, such as pancreatitis and cystic fibrosis.

Some factors that allow the number of people with diabetes to increase are population growth, aging, physical inactivity and urbanization. Diabetes of any type can cause complications in different parts of the body and can increase the overall premature death. A estimation of, that 40 million deaths, or 75% of the 53 million deaths have been suggested in 2010, were due to Non communicable diseases and injuries, including mainly of cardiovascular diseases that occupied 30%, cancers rated at 15%, chronic respiratory diseases counted 7% and diabetes at 2% 2.

In 1994 three million people in Sub-Saharan Africa (SSA) had type 2 diabetes, but that number was projected to increase by two or threefold by 2010. In 2025, it is expected that 18.7 million people will have diabetes in that region 3. Global estimate for types 2 diabetes for adult people between 20–79 years will increase to 35.5% in 2035, and enough number of people with T2D will live in that region 4.

A higher morbidity and mortality rates of type 2 diabetes in SSA countries as well as Rwanda as one of the countries are faced up and also women with Gestational diabetes Mellitus are among the highest risk population to have type 2 diabetes (See 8). According to the latest data from the WHO, non-communicable conditions account for 36% of deaths in Rwanda, where diabetes counted 1% in 2012 5. Rwanda registered almost 194,300 cases of adult diagnosed of diabetes and about 5000 diabetic related deaths was found^6^. A study conducted in 2014 in three districts shows that 544 patients received treatment for diabetes and the majority of patients had type 2 diabetes^7^.

Rwanda is one of the among few countries in SSA that have a strategic plan for NCD integrated into their public health care system Rwanda is included but the management of this chronic disease is still a problem^8^. Generally the increase of diabetes is due to either modifiable risk factors such as lack of physical activity, the use tobacco, use of alcohol, and unhealthy diets like increased fat and sodium. Some risk factors like both low quantity and quality of fruits and vegetables intake which can be controlled by intervention others are non-modifiable risk factor which are risk factor that cannot be controlled by involvement; for example: gender, age, family history, and race or to metabolic risk factors such as raised blood pressure, raised total cholesterol elevated glucose, obesity and overweight. Behaviors or modifiable risk factors can lead to metabolic or physiologic changes. Type 2 diabetes is caused by modifiable risk factors and is the most common worldwide 9.

Prevalence is the value that indicates the number of a population is affected by a medical condition. Globally, the prevalence of diabetes worldwide was 8.8 % in 2015 in the adult population and it is predicted to 10.4% in 2040 10. This is due to an increase in associated risk factors such as use of tobacco, increase of consumption of fats, sugar, alcohol, animal products, ages, family history, and diseases of pancreas. Also a decrease of physical exercises causing an inactive lifestyle is associated with obesity, diabetes, and hypertension. The economic development and urbanization lead to changing lifestyles characterized by increase in physical inactivity and increased obesity thus escalation of diabetes 11.

To plan for future health service care of citizens, policy makers must have sufficient information about the number of patients, rate and changes in prevalence over the time. This needs reliable approximation of specific variables examples age, sex, height, weight for prevalence rates and these may change due to different characteristics population and its environment and other factors. In a study conducted by International diabetes association in 2015, the prevalence of diabetes in Rwanda was about 3.16% of the population with 1,918 diabetes related deaths per year 12.

A related study conducted by Anthony Bazatsinda in 2016 (See 13), the prevalence of type 2 diabetes mellitus in patients with hepatitis C was 22.5% which was relatively high and could be due to the fact that most of patients in the study were above 40 years. This is higher compared to that of type 2 diabetes in general in Rwandan population. A report by WHO shows that in 2015 the prevalence of type 2 diabetes in Rwanda was 2.7% in female and 3.0% in male which correspond to a total of 2.8% 14.

Rwanda is a developing country that needs health and strong population physically and mentally to sustain development and this will be possible with well informed policies in health sector.

Therefore, the purpose of this work is to develop a model for predicting the prevalence of diabetes in Rwandan population, which will enable not only to predict the growth of the disease, based on the indicators of past years, but also to help decision makers to carry out preventive measures in order to reduce mortality and disability among Rwandan population. One of the methods that can help in prediction is Markov Chain Monte Carlo Method (MCMC). It is a method for forecasting by simulation the future outcome of a statistic in a mathematical model. Successive random selections form a Markov chain, the stationary distribution of which is the target distribution. It is particularly useful for the estimation of posterior distributions in complex Bayesian models. One of the common MCMC method is the Metropolis-Hastings method. In this method, we use the Metropolis Algorithm to generate a Markov chain from an arbitrary distribution 15.

The present work presents and justifies a mathematical tool to predict for long-term the behavior of type 2 diabetes in Rwanda based on MCMC which will help us to manage human life against diabetes in Rwanda. The results of prediction will help Government and decision makers to design timely intervention to cove the disease. This paper is organized as follows. The Section 2 describes Materials and methods and material, in Section 3 we identify the transition matrix using possible states of Markov chain. The numerical results and discussions are presented in section 4 while section 5 deals with concluding remarks.

## Material and Method

Diabetes had been measured using different biomarkers like Fasting Plasma Glucose (FPG), 2- hour oral glucose tolerance test (2hOGTT), and/or Hemoglobin A1c (HbA1c), which is the form of a blood pigment that carries oxygen (hemoglobin) bound to glucose. Regressions can be used to convert any prevalence data into statistical models and these models are used to estimate prevalence 14, 16. Many methods have been used to predict diabetes, see for example 17. However, the prediction of the risk for a long time is still challenging. To solve this problem, we create possible predictions using Markov Chain model instead of a single model. This model is very useful in different fields such as statistical inference and stochastic optimization process. Markov models use different discrete stages called Markov states. A subject in the stage can move from one state to another with a certain probability termed the transition probability 18. The Markov chain of prevalence of type 2 diabetes in Rwanda is obtained using the data presented in 14.

The passage process of one state to another is obtained using transition matrix calculated using different statistical methods. This work uses one of the MCMC methods: Metropolis-Hasting method introduced in 1953 to help chemists and physicists, statisticians to solve many problems^19^.

A Metropolis Hasting Method is one of MCMC methods that provides approaches to simulate a sequence of a random sample from the probability distribution from which is difficult to do a direct sampling and as more sample values are produced, the distribution of values are more closely to the desired distribution and is used to generate a proposal for the next draw from the chain. The proposal is accepted or rejected base on how well it fit with the desired distribution and according to probabilistic criteria. Since Metropolis Hasting is a Markov Chain, the proposed value for depend on only the current value through the condition distribution 22.

The algorithm of this method is clearly stated and is easy to understand so that it can help decision makers in the planning. Also Metropolis-Hastings is considered as a constructive and helpful starting point when learning about MCMC algorithms for no symmetric distribution. The Metropolis-Hasting algorithms must follow the following steps:

1. Choose a starting point;
2. Suggest a move to a new other position;
3. Decide whether to accept or reject the move under probability using the preceding information and available data;
4. If the move is accepted, move to the that position and go back to 1;
5. If the move is rejected, stay where you are and go back to 1;
6. Do it till a set number of moves have occurred, return all of the accepted positions.

Movement from one point to another is dictated by Metropolis-Hasting ratio (MHR). The normal distribution with mean *μ* and standard deviation *σ* for the current position is the good distribution and has a significant impact on convergence since it is a high chance of selecting a point near tothe most recent position than away. This means that if *P*_0_ ∼ *N* (*μ;σ*^2^) then *P*_1_ ∼ *N* (*μ;σ*^2^) where *P*_0_ and *P*_1_ are the current position and the most recent position respectively . If MHR is less than 1 the new point has a higher value of the following than the most recent sample, then accept the new proposal. If the new point has a lower posterior value than the most recent sample, then choose to accept or reject the new proposal randomly, with a probability equal to the height of both posterior values and if it is accepted, it becomes the next sample in the MCMC chain.

When the current state is t, propose a move to state s, and if the conditional probability density given s is denoted *h*(*s, t*). The Metropolis Hasting ratio is give by

(1) r(s,t)=(k(s)h(t|s))÷((k(t))h(s|t))

where r is the acceptance ratio, h the proposal distribution and k transition probability. We accept the move to s with probability

(2) α(s,t)=min(1;r(s,t))

that is, the state after the update is t with probability *α*(*s, t*), and the state after the update is s with probability 1 − *α*(*s; t*)^21^. We generated a random number *β* on [0; 1], if *β < r* accept the move and if *β ≥ r*, reject the candidate. This is the first iteration of MCMC. We do the same till there are enough samples^20^

### Identification of transition matrix

The Markov chain of type 2 diabetes prevalence in Rwanda is formed using the data presented in 14. By considering the strong relationship between type 2 diabetes and other non communicable disease or factors that can contribute to the increase of the disease, five classes of people were considered: a health subject can suffer from type 2 diabetes, overweight, obese, death and others (subjects whose lifestyle doesn't influence the type 2 diabetes). This means that the factors of obesity and overweight are considered because an obese or overweight person can suffer from diabetes. Now we can divide the population in five possible independent states of Markov chain as follow:

S - suffering from diabetes; V- overweight; B - obesity; D - dead and O - others. Let *P_ij_* be the probability of passing from state *i* to state *j*. Reffering to equation (1) the Metropolis ratio can be calculated and the acceptance of the move will be base on the following relation:

(3) $$\alpha (j|t)=\min\{1,r\}=P_{ij}$$

where *a*(*j | i*) denotes the probability of moving from state i to state j and r is acceptance ratio.


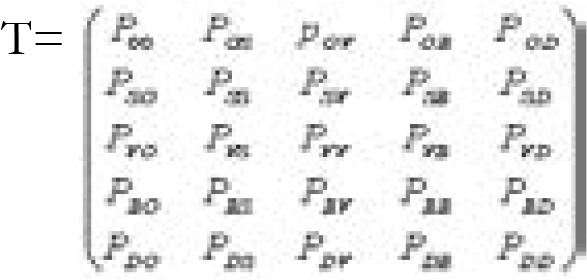


Is the transition matrix obtained using values in14, equation (1) and (2)

The move from i to j is accepted if *P_ij_* < 1, otherwise you remain in the state. With the standard normal distribution where the mean is 0 and variance of 1, the Metropolis ratio to move from the class of other subjects (O) to people suffering from diabetes (S) is calculated as follows.

(4) $$\begin{array}{l} r(S,O)=(k(S)h(O|S))÷(k(O))h(S|O)\\ r(S,O)=(k(S)h(O|S))÷(k(O))h(S|O)\\ =P(S^{2})÷P(O^{2})\\ =0:0282÷0:763882\\ =0:00134 \end{array}$$

Then the acceptance probability is *min*(1; 0:00134). Since the probability is less than one, we accept the movement with *P_os_* = 0:00134. The move from people suffering from diabetes to healthy is

**Figure 1:**
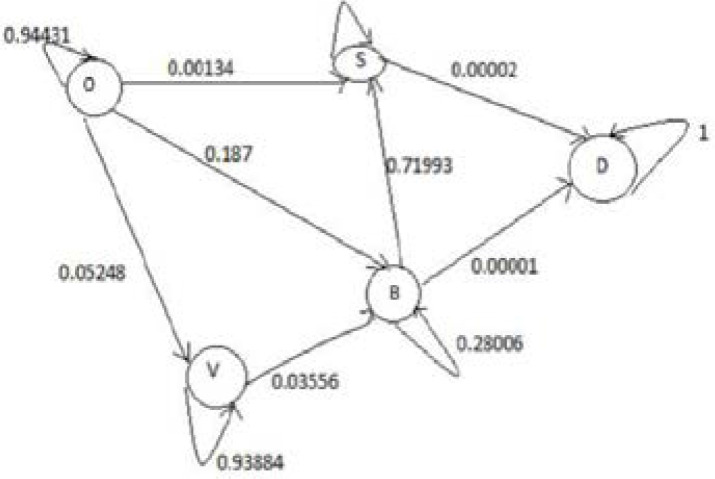
Markov Chain for type 2 diabetes in Rwanda

Hence, we accept the movement with a probability equal to zero. All other moves are calculated in the same way as done above. After calculations all states of Markov Chain for type 2 diabetes in Rwanda can be presented in the Figure 1.

From 1 the state O is non recurring state, that is, one can come into the state O only from the state O. The states might have relationship, and Markov chain end, if it contains at least one absorbing state from which the type 2 diabetic patient cannot pass into other states from that state D. This means each state someone can come to the state D, which is not possible to return from. Taking into account to the above considerations shown in the Figure 1, the transition matrix T whose rows and columns are arranged in the order O, S, V, B and D is as follows:

$$T=\left(\begin{array}{ccccc} 0.94431 & 0.00134 & 0.05248 & 0.00187 & 0\\ 0 & 0.99998 & 0 & 0 & 0.00002\\ 0 & 0.0256 & 0.93884 & 0.03556 & 0\\ 0 & 0.71993 & 0 & 0.29006 & 0.00001\\ 0 & 0 & 0 & 0 & 1 \end{array}\right)$$

Let *χ*^(0)^ be the starting point, the next point

(5) $$X^{\left(n\right)}=Trans(X^{\left(o\right)})T^{\left(n-1\right)}.$$

Then the Markov chain for patients with type 2 diabetes will have the form that is presented in Figure 1.

### Numerical results and discussion

**Figure 2:**
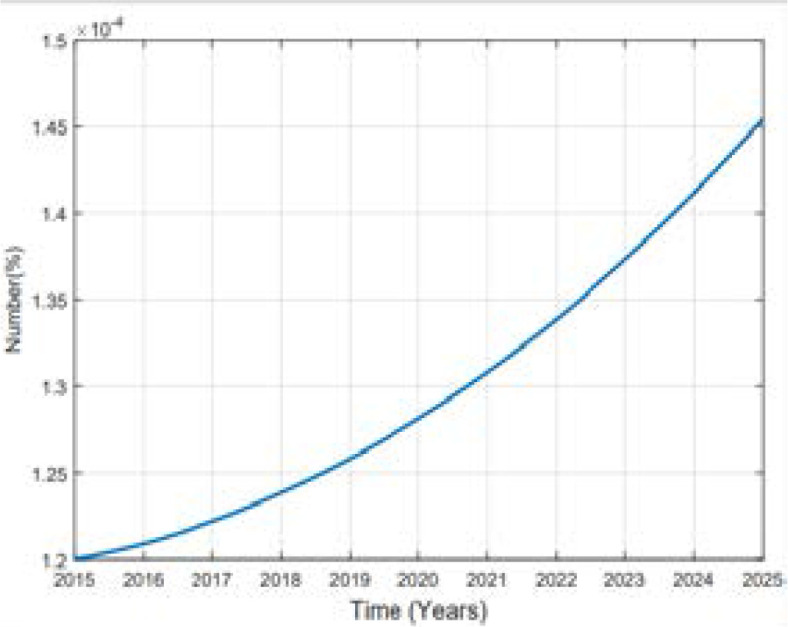
Variation of Prevalence of death

**Figure 3:**
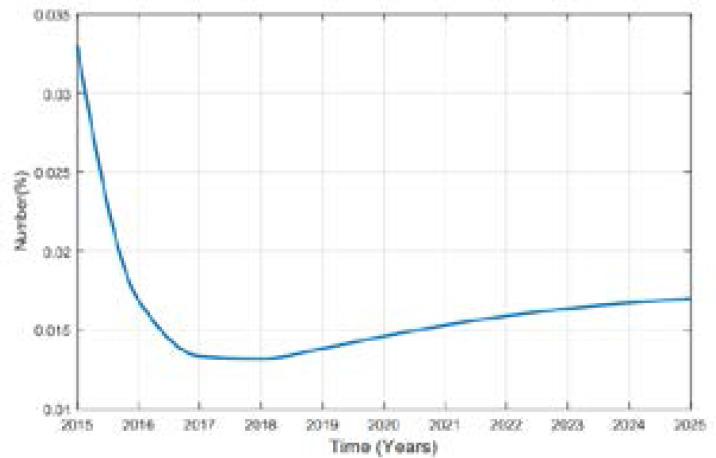
Variation of Prevalence of Obesity

**Figure 4:**
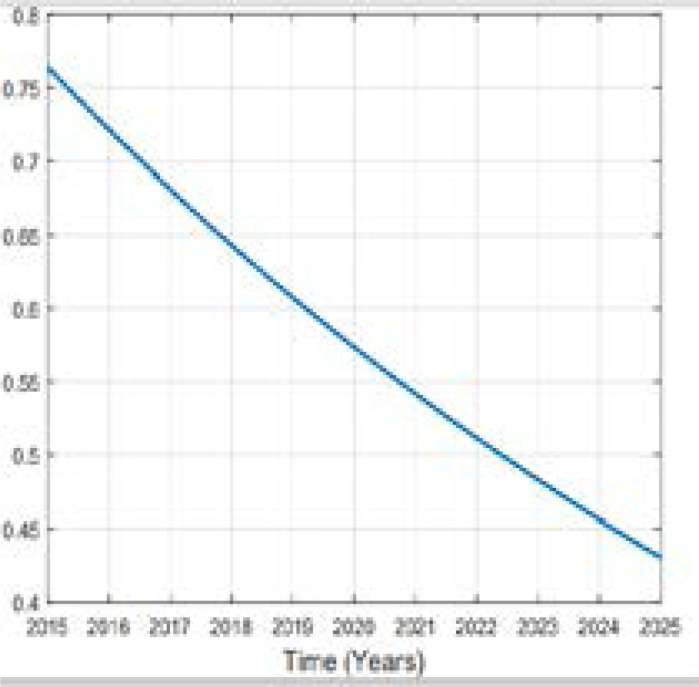
Variation of Prevalence of Health

**Figure 5:**
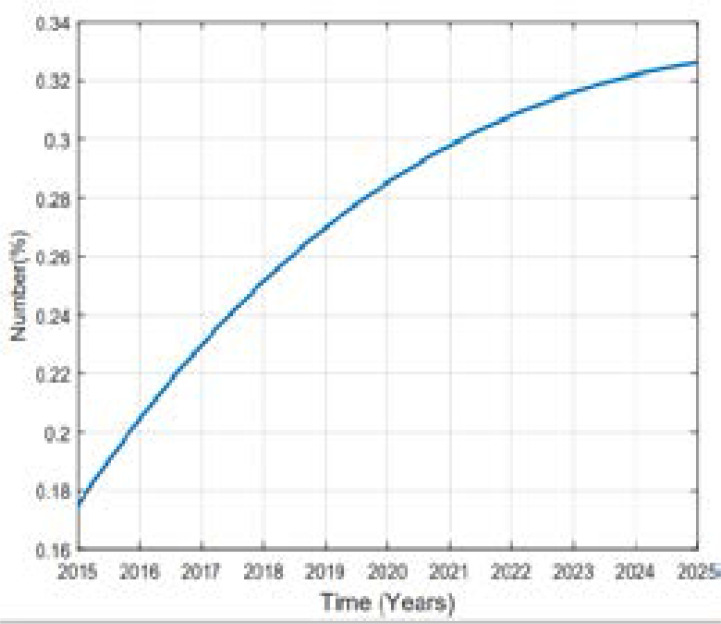
Variation of prevalence of Overweight

**Figure 6:**
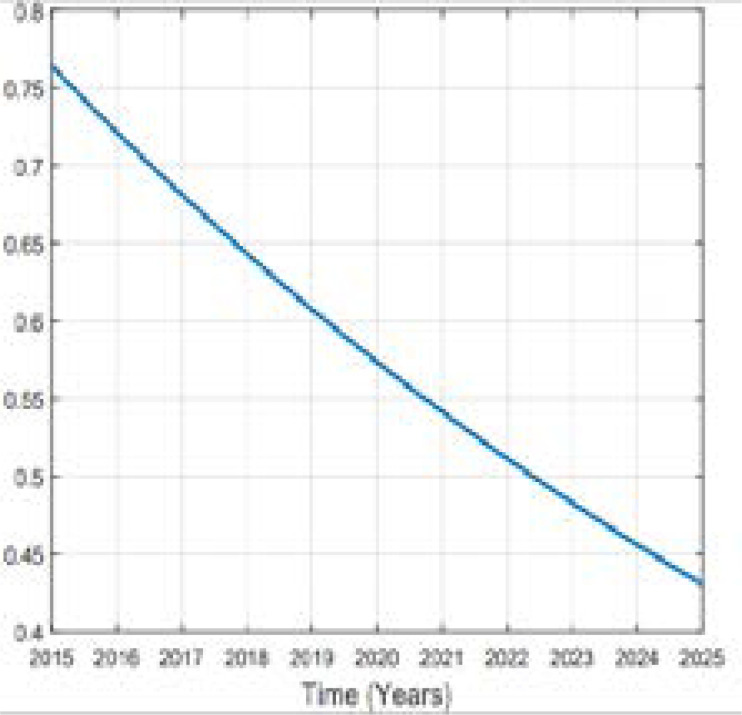
Variation of Prevalence of other subject

In this study of predicting types 2 diabetes in Rwanda, Metropolis-Hasting method is implemented and using the equation (5), codes in MATLAB package version R2018a, the numerical results are illustrated in the Figures 2, 3, 4, 5 and 6.

The predicted prevalence is 12.65% in 2020 and 22.59.02% by 2025 as it is shown in Figure 4.

Comparing the prevalence of diabetes in 2015 and 2025 it is shown that it will be multiplied by more than 8% in 10 years. A high increase between 2015 and 2025 in prevalence of type 2 diabetes in Rwanda depends on many factors. Available literature indicates that urbanization and the change in lifestyle are one of the factors. Being overweighed or obese is strongly associated to type 2 diabetes; more prevalence of them influences more prevalence of type 2 diabetes. Intervention to overcome overweight and obesity are critical ones to prevent type 2 diabetes. The prevalence of obesity will be decreased to 1.7% in 2025. The prevalence of overweight was 17.5% in 2015 and is expected to be 28.52% and 32.63% in 2025 while the prevalence of other subjects was 76.30% in 2015 and it will be 57.36% in 2020 and 43.07% in 2025. The rate of death caused by diabetes will be 0.00001 in 2025. Over the 10 years the prevalence of diabetes will be increased as well as prevalence of overweight while the one of other subjects will decrease in Rwandan people if no interventions are designed to help fight the disease.

## Conclusion

Metropolis-Hasting method can predict multiple stages of diseases in medicine. This is supported by the prediction of type 2 diabetes in Rwanda. There exist many factors that play a significant role in the development of diabetes some of them are modified and might be controlled such as lifestyle, alcohol dosage, smoking, overweight. On the other-hand, some factors are uncontrollable like age, gender, family genetic. The role of Physician doctors should not only be treatment but also prevention of the disease. The government of Rwanda needs to come up with strategies of short term and long term to prevent non communicable diseases for example sport activities and physical activities. The strategies will not doubt increase of prevalence of healthy people by 2025 there by decreasing diabetes in the country. Regular checkups should be done in urban and rural areas, health meals are recommended for better life of citizens. Most of these strategies are mentioned and available in literature 9. The combination of all these efforts makes successful measures for the diseases and their occurrence and evolution will be stopped. The use of Markov Chains Monte Carlo method (MCMCM) helps health decisions makers to predict the prevalence of type 2 diabetes in the future and take an adequate national preventive strategies and planning for the future of Rwanda.

The study has provided evidence that Metropolis-Hasting allows for better stratification and suggests avenues for future researchers to investigate other kinds of risk factors whether they can be managed for better understanding and preventing type 2 diabetes. In addition, diabetes associations must keep all individuals information on treatment basis for accurate information for secondary data use purpose instead of collecting the new data every time when they are needed for an important research. This model can be extended to include interventions.
